# *TP53* loss attenuates C1q-associated macrophage remodeling in early adenomatous polyps in a porcine FAP model

**DOI:** 10.3389/fimmu.2026.1837646

**Published:** 2026-07-13

**Authors:** Qixia Chan, Wei Liang, Tatiana Flisikowska, Friederike Ebner, Krzysztof Flisikowski

**Affiliations:** 1 Chair of Infection Pathogenesis, School of Life Science, Technical University of Munich, Freising, Germany; 2 Chair of Reproductive Biotechnology, School of Life Science, Technical University of Munich, Freising, Germany

**Keywords:** p53, Pig model, Polyposis, C1q^+^ macrophage, Tumor microenvironment

## Abstract

**Background:**

Familial adenomatous polyposis (FAP) is a hereditary condition that almost invariably leads to colorectal cancer. While *TP53* inactivation is well established as a late event in the adenoma-to-carcinoma sequence, its functional role during the early stage of colorectal polyp development remains unclear.

**Methods:**

Three lines of FAP pigs were generated: *APC^1311/+^*, *APC^1311/+^*/*TP53^+/-^*, and *APC^1311/+^*/*TP53^-/-^*. Colonic polyp development was evaluated by colonoscopy at 3 and 9 months of age. Mucosal goblet cell and mucin content were assessed using periodic acid-Schiff/Alcian blue (PAS-AB) staining. Immunohistochemistry for Ki67 and IBA1 was performed to assess epithelial proliferation and macrophage/monocyte-like cell infiltration, respectively. Quantitative PCR was used to measure mRNA expression of macrophage/monocyte - and complement-related genes, including *CCL2*, *FCGR3A*, *ITGAM*, and members of the C1q family.

**Results:**

Macroscopic adenomatous polyp burden and growth dynamics were comparable across genotypes. However, *APC^1311/+^*/*TP53^-/-^* pigs exhibited significantly reduced epithelial proliferation within polyp crypts, as indicated by decreased Ki67^+^ expression. Furthermore, immunohistochemistry revealed a significant reduction of stromal IBA1^+^ macrophage/monocyte-like cells in polyps from *APC^1311/+^*/*TP53^-/-^* animals. Consistently, expression levels of *CCL2*, *ITGAM*, *FCGR3A*, and complement components *C1QB* and *C1QC* were significantly downregulated.

**Conclusions:**

Loss of *TP53* did not accelerate early-stage adenomatous polyp progression in the FAP pig model. However, *TP53* deficiency was associated with significant alterations in the polyp microenvironment, particularly characterized by reduced C1q-associated macrophage remodeling and diminished expression of macrophage/monocyte- and complement-related genes. These findings suggest a role for *TP53* in modulating the inflammatory tumor microenvironment during colorectal tumorigenesis.

## 1 Introduction

Familial adenomatous polyposis (FAP), a severe hereditary disease caused by germline mutations in the adenomatous polyposis coli (*APC*) gene, is characterized by a high risk of colorectal cancer (CRC) development (1). In humans, somatic *APC* gene mutations are considered the initiating event in the vast majority of sporadic CRC cases (2). These mutations lead to aberrant activation of the *Wnt*/β-catenin signaling pathway, thereby facilitating the conversion of normal colorectal epithelial cells into adenomas (3, 4). In FAP, a wide spectrum of germline *APC* mutations has been identified, and these mutations are associated with distinct clinical phenotypes, including variation in disease severity and polyp burden (5). The human *APC^1309^* results in a truncated APC protein and drives early, severe, profuse colorectal polyposis (6). In pigs, the *APC^1311^* mutation, orthologous to human *APC^1309^*, closely recapitulates human FAP including polyp location and disease severity (7, 8). In addition, pigs share important anatomical and physiological similarities of the gastrointestinal tract with humans, enhancing their value as a model for studying intestinal diseases. In contrast, commonly used *APC* mutant mouse models have provided important insights into FAP pathogenesis but predominantly develop tumors in the small intestine rather than the colon and rectum (9, 10). This lesion distribution limits their utility for studying early colorectal tumorigenesis.

The development of CRC is widely recognized as a multistep evolutionary process driven by the sequential accumulation of mutations and/or inactivations in tumor suppressor genes (3, 11). In addition to *APC* alterations, the tumor suppressor p53 (encoded by *TP53*) plays a pivotal role in maintaining intestinal epithelial homeostasis by regulating cell cycle progression, DNA repair, and apoptosis (12). In the colonic epithelium, rapid and continuous cell turnover is essential for tissue renewal (13). p53 acts as a critical safeguard against uncontrolled proliferation by limiting the expansion of genetically damaged cells. Mutated or inactivated p53 impairs these regulatory mechanisms, leading to increased epithelial proliferation and contributing to colorectal tumor initiation and progression (12–14). In addition, p53 deficiency is associated with changes in epithelial remodeling and tumor microenvironment (TME), involving goblet cells secretion (15) and macrophage differentiation (16).

The p53 plays an important role in cell homeostasis, including immune homeostasis, through regulation of both innate and adaptive immune signaling pathways (12). Additionally, p53 suppresses inflammation (17), mainly by modulating the tumor secretion of cytokines (18). In response to cellular stress, p53 activation induces the secretion of pro-inflammatory chemokines, including *CCL2*, a key mediator of monocyte/macrophage recruitment (19). Following recruitment to the TME, macrophages/monocytes undergo context-dependent phenotypic reprogramming that shapes their pro-tumor or anti-tumor functional properties (20). Recent single-cell transcriptomic studies have identified a distinct and highly immunosuppressive subset of tumor-associated macrophages (TAMs) characterized by expression of markers such as *ITGAM* (CD11b), *FCGR3A* (CD16), and members of the C1q family (*C1QA*, *C1QB*, and *C1QC*) (21, 22). *ITGAM* serves as a critical integrin and complement receptor (CR3), facilitating myeloid cell adhesion, migration, and phagocytosis (23). Concurrently, *FCGR3A* encodes a low-affinity Fc-gamma receptor that mediates antibody-dependent cellular cytotoxicity (ADCC) and the robust phagocytosis of immune complexes (24). *ITGAM* and *FCGR3A* are functionally specialized for cellular clearance, while they are strongly linked to immunosuppression and tumor progression in the TME (21–24). C1q, the recognition subcomponent of the classical complement pathway, is predominantly produced and secreted by macrophages (25). Accumulating evidence indicates that C1q^+^ TAMs are enriched across multiple malignancies, and their presence within the TME strongly correlates with tumor progression and metastasis. Functionally, these macrophages contribute to the establishment of an immunosuppressive microenvironment by promoting epithelial-mesenchymal transition (EMT), enhancing tumor cell proliferation, and attenuating cytotoxic T-cell activity via the production of complement components that modulate immune cell infiltration (26, 27).

We previously generated transgenic pigs carrying the *APC^1311/+^* mutation (7) and a *TP53* knockout (*TP53-*KO) allele (28). Notably, our earlier work revealed increased *TP53* mRNA expression in adenomatous polyps (*APC^1311/+^* pigs) (29), suggesting a potential role for p53 during early polyp development. However, while the role of *TP53* alterations in the late stages of CRC progression has been extensively investigated (3, 11), its contribution to the early stages of *APC*-driven polyp development remains unclear. In this study, we further explore the functional contribution of p53 in early adenoma formation. *APC^1311/+^* and *TP53-*KO lines were crossed to generate compound mutant pigs. These models enable a systematic evaluation of the effects of *TP53* loss on the TME in adenomatous polyps.

## 2 Materials and methods

### 2.1 Ethical statement

All pig experiments were performed at Technical University of Munich (TUM) following the principles outlined in the European Convention for the Protection of Vertebrate Animals used for Experimental and Other Scientific Purposes and the German Animal Welfare Law. Ethical approval was granted by the Committee on Animal Health and Care of the local government body of the state of Upper Bavaria (Permission No. 55.2-2532.Vet_02-18-33).

### 2.2 Animal models

The porcine *APC^1311/+^* model, carrying a truncating mutation in the *APC* gene that results in hundreds to thousands of polyps in the colon and rectum (7), and the *TP53-*KO model, generated using a loxP-STOP-loxP transcriptional termination cassette to silence *TP53* expression (28), were used to generate *APC^1311/+^/TP53^+/-^* and *APC^1311/+^/TP53^-/-^* pigs. These genotypes were analyzed to model the stepwise inactivation of p53 and to determine whether 50% loss of p53 functionality is sufficient to accelerate the *APC*-driven oncogenic phenotype. For genotyping, genomic DNA was extracted from ear biopsies using the GenElute Mammalian Genomic DNA Miniprep Kit (Sigma-Aldrich, USA) according to the manufacturer’s protocol. The genotyping PCR for *APC* and *TP53* was performed using GoTaq G2 DNA polymerase (Promega, USA) using primer pairs: Fwd: (5' gagcaacggctacaatca 3'), Rev: (5' tgagcaccactctttgatgg 3') and the following PCR program: 94 °C for 2 min; 40 × [94 °C for 20 s, 59 °C for 15 s, 72 °C for 45 s]; 72 °C for 1 min, and Fwd: (5' tgaggaatttgtatgccaagg 3'), Rev: (5' ttccaccagtgaatccacaa 3') detected by following conditions: 95 °C for 5 min; 30 × [95 °C for 30 s, 58 °C for 30 s, 72 °C for 90 s]; 72 °C for 5 min, respectively (7, 28). Representative genotyping results are shown in **Supplementary Figure 1**.

### 2.3 Endoscopic assessment of polyps

To assess polyp development, animals underwent colonoscopic examination (KARL STORZ endoscopy system, Germany) at 3 and 9 months of age. For the procedure, pigs were anesthetized by intramuscular administration of ketamine (Ketamidor^®^, 100 mg/mL; Richter Pharma AG, Wels, Austria; 20 mg/kg BW) in combination with azaperone (Stresnil^®^, Elanco Animal Health, Greenfield, IN, USA; 2 mg/kg BW). During colonoscopy, the colorectal mucosa was examined for the presence of polypoid lesions, and the number and size of detected polyps were recorded. Several animals had polyp burden exceeding 200 lesions, in some cases rendering precise quantification impractical. For standardization and clarity of presentation, a threshold of 200 polyps was therefore applied.

### 2.4 Tissue sampling

All pigs were housed and managed at the TUM Animal Research Center (ARC). At 9 months of age, animals were sacrificed for sample collection. For euthanasia, pigs were first anesthetized via intramuscular injection of ketamine (Ketamidor^®^, 100 mg/mL; 20 mg/kg body weight; Richter Pharma AG, Wels, Austria) in combination with azaperone (Stresnil^®^, 2 mg/kg body weight; Elanco Animal Health, Greenfield, IN, USA). Following anesthesia, animals were euthanized by bolt shooting followed by exsanguination. Polyp and normal mucosa (NM) samples from *APC^1311/+^* (n = 16), *APC^1311/+^/TP53^+/-^* (n = 10) and *APC^1311/+^/TP53^-/-^* (n = 10) pigs were collected and stored at -80 °C until further analysis. Polyp samples for histological and immunohistochemical analysis were rinsed in cold phosphate-buffered saline (PBS) and fixed in 4% paraformaldehyde (Sigma-Aldrich, USA) for 24 hours at room temperature. After fixation, specimens were transferred into 70% ethanol (Sigma-Aldrich, USA) and then embedded in paraffin for further processing.

### 2.5 Histology and immunohistochemistry

Serial sections (3.5 µm) of paraffin specimens were prepared using a MICROM HM 355 S rotary microtome (MICROM GmbH, Germany) for subsequent processing (30). Periodic Acid-Schiff-Alcian Blue (PAS-AB) staining was performed as previously reported (31) with minor modifications. Briefly, deparaffinized sections were stained with 1% Alcian blue (Sigma-Aldrich, USA) prepared in 3% acetic acid (pH 2.5; AppliChem GmbH, Germany), oxidized with periodic acid (Sigma-Aldrich, USA), incubated with Schiff’s reagent (Sigma-Aldrich, USA), counterstained with hematoxylin (Sigma-Aldrich, USA), dehydrated through graded ethanol and xylene, and mounted. AB^+^ blue acidic signals were quantified from 10 selected regions containing aberrant crypts at the same magnification from each polyp using ImageJ 1.48v (National Institutes of Health, Bethesda, MD, USA). PAS^+^-AB^+^ purple acidic/neutral mixed mucin signals were assessed from 10 regions containing aberrant crypts within a polyp at the same magnification using a semi-quantitative method: PAS^+^-AB^+^ areas were first quantified using ImageJ, and the final results were then manually reviewed to ensure accuracy (32). For protein expression analysis, immunohistochemistry (IHC) was performed to detect Ki67 (cell proliferation) and IBA1 (macrophage/monocyte marker) In brief, sections were incubated with the following primary antibodies: Anti-Ki67 (clone SP6; 1:100 dilution; Invitrogen, MA5-14520, USA) and Anti-IBA1 (rabbit polyclonal; 1:2000 dilution; Wako Fujifilm, 019-19741, Japan); Signal detection was achieved using a horseradish peroxidase (HRP)-conjugated secondary antibody (Goat Anti-Rabbit IgG-HRP; 1:200 dilution; Southernbiotech, 4030-05, USA) and a biotinylated secondary antibody (Goat Anti-Rabbit IgG; 1:100 dilution; Vector, BA-1000, USA), respectively (33). Ki67^+^ and IBA1^+^ cells were quantified by counting positive (stained cells: brown) and negative (unstained cells: blue) cells in 10 aberrant crypts and stroma selected from each polyp using ViewPoint 1.0.0.9628 (PreciPoint, Germany). The specificity of both antibodies was established in our previous studies (33, 34).

### 2.6 RNA extraction and quantification

Total RNA was isolated from NM and polyps using a Monarch Total RNA Miniprep Kit (New England Biolabs, USA) following the manufacturer’s protocols. RNA was reverse transcribed into cDNA using the LunaScript RT Master Mix Kit (New England Biolabs, USA). Real-time quantitative PCR (qPCR) was performed using qPCRBIO SyGreen Mix Lo-ROX (Biosystems, UK) on a QuantStudio 5 Real-Time PCR System (Thermo Fisher Scientific, USA) with default thermal cycling parameters. Reactions were conducted in a 10 µl volume. Specific primers (**Supplementary Table 1**) were selected from *Sus scrofa* (Sscrofa11.1) nucleotide sequences retrieved from the GenBank database. Samples were assayed in triplicate, and relative expression levels were normalized to *GAPDH* (**Supplementary Table 1**). Relative gene expression was calculated by the ΔΔCt method (35).

### 2.7 Statistical analysis

Data are expressed as mean ± standard error of the mean (SEM). Normal distribution and homogeneity of variance were assessed using Shapiro-Wilk and Levene’s tests, respectively. For comparisons among three groups, one-way ANOVA was used, followed by Tukey’s *post hoc* test for multiple comparisons. For two-group comparisons, the Independent Samples *t*-test was employed. When data were not normally distributed, the Kruskal-Wallis *H* test or Mann-Whitney *U* test was used accordingly. All statistical analyses were performed using SPSS software version 22.0 (IBM Corp., USA). Statistical significance was set at *P* < 0.05. Data visualization was performed using GraphPad Prism 8.0.

## 3 Results

### 3.1 p53 loss does not accelerate the malignant progression of polyps in *APC^1311/+^* mutant pigs

**Figure 1:**
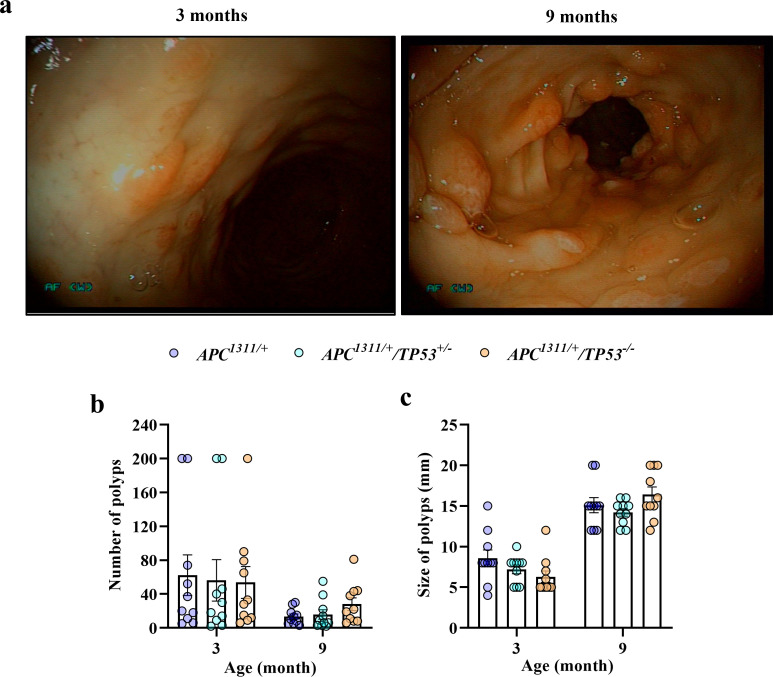
Summary of *TP53*-dependent colon polyposis in porcine FAP models. **(a)** Representative macroscopic images of colon polyps at 3 and 9 months of age were shown from *APC^1311/+^/TP53^-/-^* pig. **(b)** The number of polyps at 3 and 9 months of age counted in the colon of *APC^1311/+^*, *APC^1311/+^/TP53^+/-^* and *APC^1311/+^/TP53^-/-^* pigs. **(c)** The size of polyps at 3 and 9 months of age in *APC^1311/+^* (blue dots), *APC^1311/+^/TP53^+/-^* (green dots) and *APC^1311/+^/TP53^-/-^* (orange dots) groups. The Kruskal-Wallis *H* test and Dunn’s *post hoc* test were applied to non-normally distributed data. Data are presented as mean ± SEM (n = 10 pigs).

To investigate the effect of p53 loss on polyp development in *APC^1311/+^*, *APC^1311/+^/TP53^+/-^* and *APC^1311/+^/TP53^-/-^* mutant pigs, colonoscopy was performed at 3 and 9 months of age, no detectable differences among the three genotypes were observed (**Figure 1a**; **Supplementary Figure 1**). At 3 months of age, the total number of polyps was comparable across the three genotypes, with mean counts ranging from 53 to 62 (**Figure 1b**). Each group exhibited considerable inter-individual heterogeneity. At the 9-month of age, a decline in polyp number across all genotypes was observed (**Figure 1b**). The *APC^1311/+^/TP53^-/-^* animals showed a marginally higher number of polyps at 9 months than the other two groups, although this difference did not reach statistical significance (**Figure 1b**). The average polyp size increased from 3 to 9 months of age, with the maximum diameter reaching 20 mm; however, no significant difference in polyp size among the genotypes was observed (**Figure 1c**).

### 3.2 p53 loss alters epithelial proliferation and macrophage/monocyte-lineage cell infiltration in polyps

**Figure 2:**
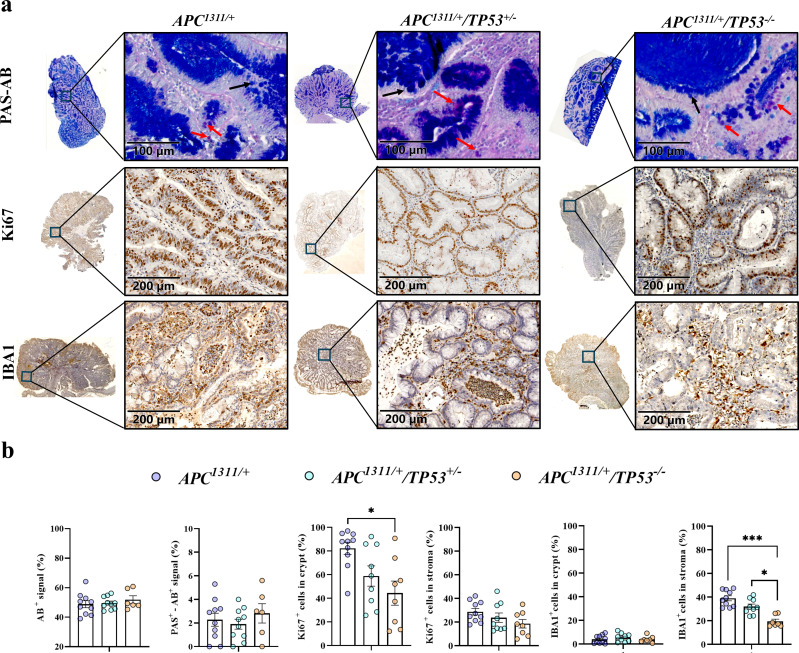
Histopathological and immunohistochemical characterization of adenomatous polyps in FAP pig lines. **(a)** Representative histological (PAS-AB) and immunohistochemical (Ki67 and IBA1 staining) images with corresponding statistical quantifications for *APC^1311/+^* (blue dots), *APC^1311/+^/TP53^+/-^* (green dots) and *APC^1311/+^/TP53^-/-^* (orange dots) groups. Scale bar = 100 or 200 μm. **(b)** Quantification of AB^+^ signal (blue: black arrow), semi-quantification of PAS^+^-AB^+^ signal (purple: red arrow), and quantification of Ki67^+^ and IBA1^+^ cells. For AB^+^ staining, each data point represents the mean of positive signal quantified from 10 regions containing aberrant crypts within a polyp at identical magnification using ImageJ. For PAS^+^AB^+^ staining, each data point represents the mean of positive signal semi-quantified from 10 regions containing aberrant crypts within a polyp at identical magnification using ImageJ and manual method to ensure accuracy. For immunohistochemical analyses, Ki67^+^ epithelial cells and IBA1^+^ macrophages (positive cells: brown staining) were quantified in crypt and stromal compartments. Each data point represents the mean number of positive cells determined by counting stained (brown) and unstained (blue) cells in 10 aberrant crypts and corresponding stromal regions per polyp using ViewPoint. Normal mucosa served as the reference control across all staining assays. One-way ANOVA followed by Tukey’s HSD *post hoc* test for normally distributed data with homogeneous variances. For non-normally distributed data, the Kruskal-Wallis *H* test followed by Dunn’s *post hoc* test was employed for pairwise comparisons. Data are presented as mean ± SEM (n = 4–10 pigs, 1–3 polyps selected for staining from each pig); ^*^ means *P* < 0.05, ^***^ means *P* < 0.001.

Since p53 knockout did not affect polyp number, we next asked whether it influences polyp histopathology. First, we focused on assessing the mucosal barrier integrity by using PAS-AB staining. This analysis revealed no significant differences among polyps from the three groups (**Figures 2a, b**), suggesting that p53 deficiency does not markedly alter mucus production or epithelial barrier in early adenomatous polyps.

Epithelial proliferative activity was subsequently assessed by Ki67 expression. Quantitative analysis revealed a significantly greater number of Ki67^+^ cells within the polyp crypts of *APC^1311/+^* pigs compared to *APC^1311/+^/TP53^-/-^* pigs (*P* < 0.05; **Figures 2a, b**). No significant differences in Ki67^+^ cells in the stroma among the genotypes were detected (**Figures 2a, b**).

We further studied IBA1^+^ macrophage/monocyte frequency, an important component of the TME and a predominant immune cell population that contributes to immunosuppressive signaling in colon cancer (36). Immunohistochemistry revealed a significantly reduced frequency of IBA1^+^ macrophage/monocyte in polyp stroma of *APC^1311/+^/TP53^-/-^* pigs compared to *APC^1311/+^* (*P* < 0.001; **Figure 2b**) and *APC^1311/+^/TP53^+/-^* pigs (*P* < 0.05; **Figure 2b**). In contrast, no significant differences in IBA1^+^ cell frequency in polyp crypts among the three groups were observed (*P* > 0.05; **Figures 2a, b**).

### 3.3 p53 loss suppresses macrophage/monocyte-associated chemotactic and complement gene expression

To unravel the molecular profile driving the observed stromal macrophage depletion, we investigated the expression of pro-inflammatory and complement-associated genes. The mRNA expression of the key mediator of monocyte/macrophage recruitment: *CCL2*; macrophage functional receptors: *FCGR3A* and *ITGAM*; and complement system components: *C1QA*, *C1QB* and *C1QC* was studied. The *APC^1311/+^* and *APC^1311/+^/TP53^+/-^* pigs were pooled, as no phenotypic differences between these genotypes were found.

**Figure 3:**
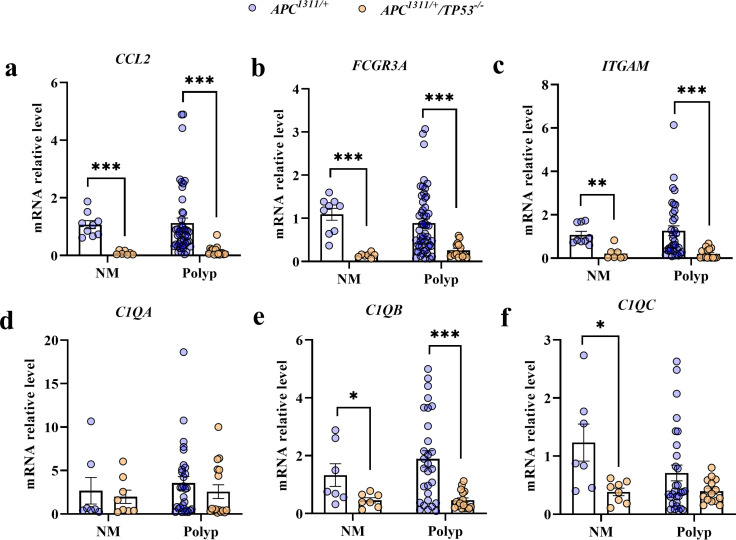
qPCR analysis of macrophage- and complement-related genes (**(a)** *CCL2*, **(b)** *FCGR3A*, **(c)** *ITGAM*, **(d)** *C1QA*, **(e)** *C1QB*, f. *C1QC*) in normal mucosa and polyp from *APC^1311/+^* (blue dots) and *APC^1311/+^/TP53^-/-^* (orange dots) pigs. The Independent Samples *t*-test was used for normally distributed data, while the Mann-Whitney *U* test was applied for non-normally distributed data. Data are presented as mean ± SEM (n = 4–16 pigs, 1–3 polyps were selected for qPCR from each pig); ^*^ means *P* < 0.05, ^**^ means *P* < 0.01, ^***^ means *P* < 0.001.

Transcript levels of *CCL2* (**Figure 3a**), *FCGR3A* (**Figure 3b**), and *ITGAM* (**Figure 3c**) were significantly downregulated in both normal mucosa and polyps of *APC^1311/+^/TP53^-/-^* pigs compared with *APC^1311/+^* pigs. Among the complement system components, the *C1QA* (**Figure 3d**) expression was similar between genotypes in both normal mucosa and polyps, whereas *C1QB* expression was significantly decreased in *APC^1311/+^/TP53^-/-^* pigs compared to *APC^1311/+^* pigs in both normal mucosa (*P* < 0.05) and polyps (*P* < 0.001) (**Figure 3e**), and *C1QC* expression was decreased in the normal mucosa (*P* < 0.05), but not in polyps (**Figure 3f**), suggesting *TP53* loss induced a suppression of macrophage-associated immune responses in TME.

## 4 Discussion

Sporadic CRC often originates from adenomatous polyps (37, 38). The transition from adenomatous polyps to malignant carcinomas typically requires additional genetic alterations, with *TP53* inactivation being a hallmark of late-stage progression in human patients (3). Building on our previous findings demonstrating elevated *TP53* expression in early adenomatous polyps (29), we generated *APC*/*TP53* knockout pigs to elucidate the functional role of p53 in the initiation of colorectal polyposis in FAP pigs. The loss of *TP53* expression in the *TP53^-/-^* pigs has been comprehensively demonstrated in several of our previous studies (28, 39–41).

More importantly, among the persistent polyps at 9 months, we found p53 inactivation did not significantly accelerate their morphological or malignant progression in FAP pigs, consistent with the canonical CRC model in which p53 exerts its functional impact predominantly in later stages of tumor progression (42). As no macroscopic phenotypic changes were observed in the polyps among pig lines, we further investigated the pathological modifications and immune remodeling in the TME. Our results suggest that *TP53* loss suppresses epithelial proliferation in early polyps. This finding is distinct from the view that *TP53* loss drives the transition from the late stage of adenoma to malignant carcinoma (3). *TP53* loss typically drives tumor progression by promoting cell proliferation (43). However, a previous study revealed that to sustain cell proliferation, p53-deficient cells exhibit a heightened reliance on certain survival factors, such as *TERT* or specific kinases; disruption of these dependencies drives them into growth arrest more rapidly than wild-type counterparts (44). Accordingly, remodeling of the TME may perturb key survival signals, thereby contributing to reduced epithelial cell proliferation in polyps in the context of p53 loss. Colorectal tumorigenesis is accompanied by substantial infiltration of immune cells (45, 46), with macrophages representing a highly plastic cell population capable of exerting both pro- and anti-tumorigenic effects (22, 47–49). Therefore, characterization of macrophage-associated immune infiltration is important for understanding the development of FAP-related colorectal lesions. In the present study, inactivation of *TP53* in *APC*-driven polyps was associated with a marked reduction in IBA1^+^ macrophage-associated infiltration, suggesting that p53 may contribute to the regulation of chemokine-driven recruitment and the maintenance of local macrophage-associated cell survival in early adenomas. Furthermore, the decrease in macrophage-associated cell abundance may disrupt early immunomodulatory signals, potentially shifting the TME balance and influencing adenoma progression. These findings align with prior evidence that p53 regulates immune cell recruitment (50, 51) and suggest a previously unrecognized role for p53 in shaping the macrophage-associated cell compartment of the early colonic TME.

Analysis of the molecular mechanisms driving macrophage-related cell depletion revealed a pronounced downregulation of macrophage-associated genes governing chemotaxis and immune activation, including *CCL2*, *ITGAM*, and *FCGR3A*. The reduction of *CCL2*, a key chemokine mediating monocyte/macrophage recruitment to the tumor stroma (19, 52), provides a plausible molecular explanation for the impaired infiltration of IBA1^+^ macrophages/monocytes in the p53-deficient polyp microenvironment. Concurrently, decreased expression of *ITGAM* (CD11b) and *FCGR3A* (CD16), established markers of macrophage abundance and functional engagement, highlights a diminished macrophage presence and activity within the TME, which are processes known to influence tumor initiation and pro-tumorigenic macrophage function (53, 54). In parallel, complement components *C1QB* and *C1QC* were downregulated, reflecting suppression of innate immune surveillance pathways essential for the clearance of stressed or transformed cells (55). Given that macrophages can both promote tumor progression and facilitate immune evasion (56), their depletion alongside reduced complement activity indicates that *TP53* loss disrupts early immune-mediated tumor remodeling, demonstrating that macrophage-related reprogramming is not merely a late-stage consequence, but an early event in tumor progression.

This study has inherent limitations. Human studies have shown that IBA1 can mark not only monocytic/histiocytic cells but also dendritic cell–lineage populations (57). In pigs, however, IBA1 is widely accepted as a marker of the mononuclear phagocyte system, and we therefore interpreted IBA1-positive cells primarily as macrophage/monocyte-lineage cells, while recognizing potential overlap with dendritic cell–related populations (33, 58–60). Further resolution of myeloid heterogeneity, including DC-associated (*IRF8*, *CD40*, *CD80*, *CD86*) and angiogenesis-related (*VEGFA*, *FGF2*, *PROK2*, *MMP9*) programs, will benefit from future studies using porcine-validated markers and high-resolution approaches such as scRNA-seq or spatial transcriptomics.

Despite these limitations, our findings suggest that *TP53* inactivation attenuates macrophage-related recruitment and innate immune surveillance in early adenomas, potentially depriving the C1q-macrophage-to-T cell immunosuppressive crosstalk necessary for tumor immune evasion. Consequently, this altered immune landscape sets the stage for the later transition from adenoma to malignant carcinoma, reinforcing its canonical role in the late stages of colorectal tumorigenesis.

## 5 Conclusion

In early FAP, *TP53* loss does not accelerate polyp progression but profoundly reshapes the tumor microenvironment, suppressing epithelial cell proliferation and depleting macrophage infiltration. These results indicate that p53 inactivation initiates immune and stromal remodeling, which may mechanistically explain the insufficient acceleration of CRC initiation upon p53 loss in FAP pigs.

## Data Availability

The original contributions presented in the study are included in the article/**Supplementary Material**. Further inquiries can be directed to the corresponding author.
