# Supplementary material for: TP53 loss attenuates C1q-associated macrophage remodeling in early adenomatous polyps in a porcine FAP model
Source: Front Immunol. 2026 Jul 13;17:1837646. doi: 10.3389/fimmu.2026.1837646 (PMC13402134; doi:10.3389/fimmu.2026.1837646)
Supplement: Supplementary file 1 [file DataSheet1.docx]

***TP53* loss attenuates *C1Q* - associated macrophage remodeling in early adenomatous polyps in a porcine FAP model**

**Qixia Chan^1^, Wei Liang^1^, Tatiana Flisikowska^2^, Friederike Ebner^1^, Krzysztof Flisikowski^1*^**

^1^Chair of Infection Pathogenesis, School of Life Science, Technical University of Munich, Freising, Germany.

^2^Chair of Reproductive Biotechnology, School of Life Science, Technical University of Munich, Freising, Germany.

*** Correspondence**

Krzysztof Flisikowski

[krzysztof.flisikowski@tum.de](mailto:krzysztof.flisikowski@tum.de)

**Supplementary Table 1.** qRT-PCR primer sequences.

| **Primer name** | **Sequence (5′–3′)** |
| --- | --- |
| *CCL2*_F | aagaagatctcgatgcagcg |
| *CCL2*_R | caggtggcttatggagtcct |
| *FCGR3A*_F | agtgacccattgcgactaga |
| *FCGR3A*_R | gaaaacttcttccccatgcca |
| *ITGAM*_F | cagcggatgaaggagtttgt |
| *ITGAM*_R | ctccccagcagctgtcttat |
| *C1QA_*F | ggctggctggtgatcatgat |
| *C1QA_*R | cggatgccagtctgaaagg |
| *C1QB_*F | tgatggcatacctgggaccc |
| *C1QB_*R | gccgacttttcctggaatcc |
| *C1QC_*F | ctactgctgcttctactggc |
| *C1QC_*R | cttctcacccttgggtccac |
| *GAPDH*_F | ttcacgaccatggagaaggc |
| *GAPDH*_R | ggttcacgcccatcacaaac |

**
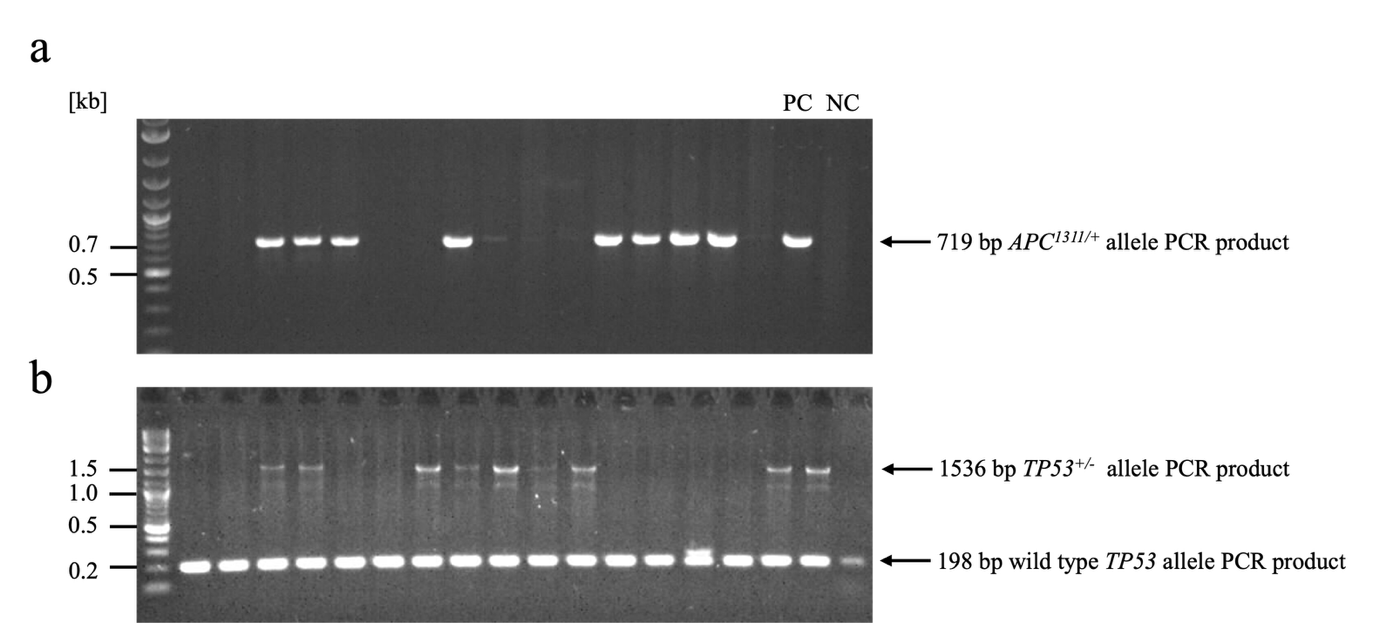
**

**Supplementary Figure 1.** Agarose gel electrophoresis of PCR-based genotyping for *APC1311* (**a**) and *TP53+/-* (**b**) mutant pigs. Genomic DNA was isolated from ear tissue, and the expected PCR amplicon size is indicated in the figure. PC – positive control, NC – negative control.

3 months


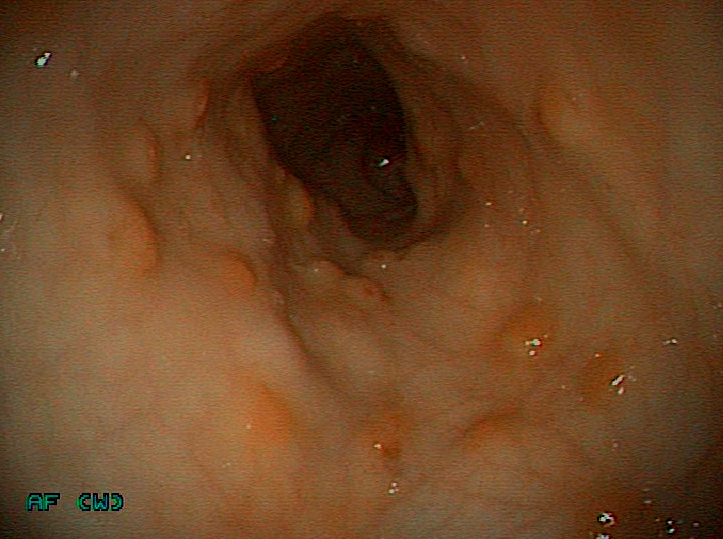

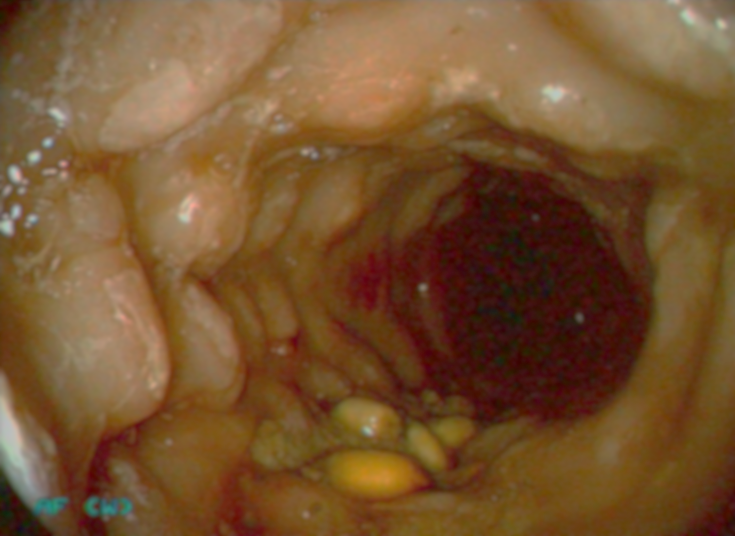

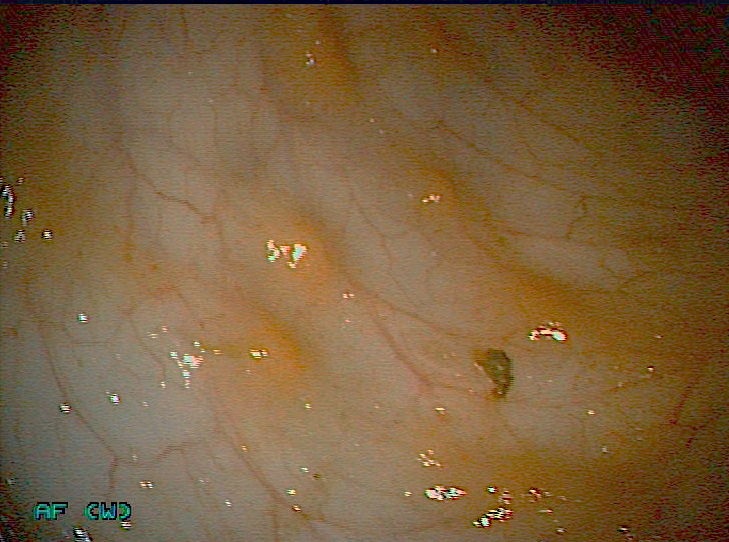

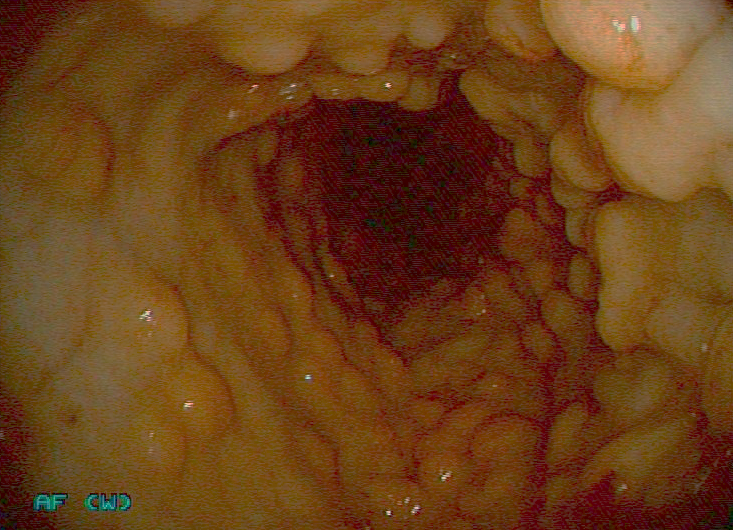


9 months

*APC^1311/+^*

*APC^1311/+^ / TP53^+/-^*

**Supplementary Figure 2.** Representative colonoscopy images from *APC^1311/+^* and *APC^1311/+^/TP53^+/-^* pigs.


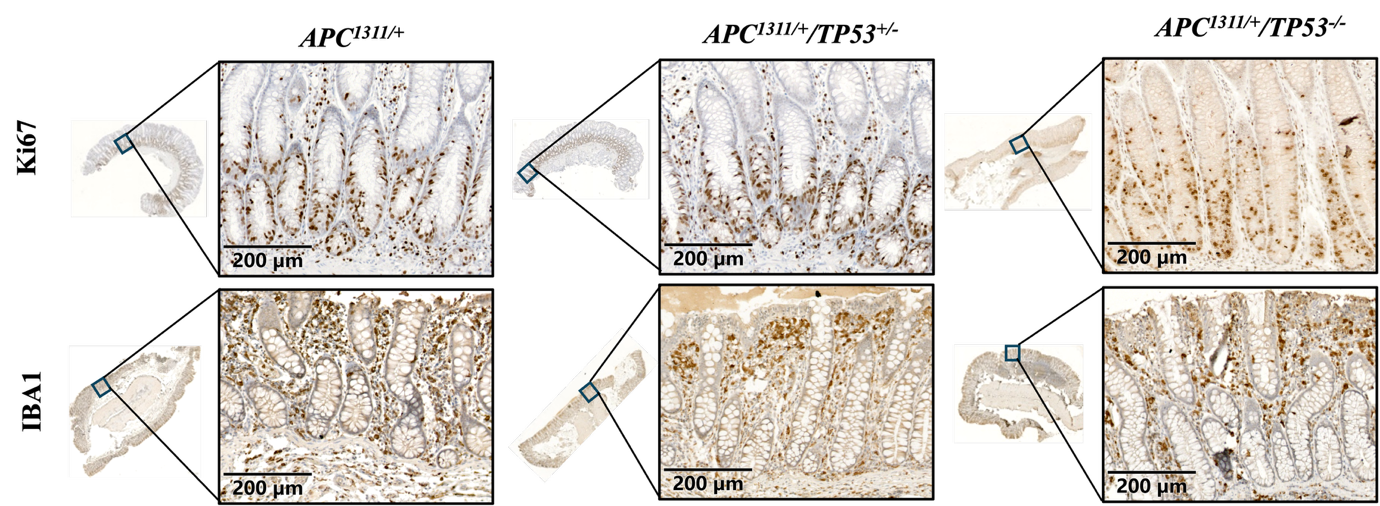


**Supplementary Figure 3.** Representative immunohistochemical images for Ki67 and IBA1 staining of normal mucosa in FAP pig lines.
